# Long‐term survival benefit of upfront chemotherapy in patients with newly diagnosed borderline resectable pancreatic cancer

**DOI:** 10.1002/cam4.1104

**Published:** 2017-06-21

**Authors:** Bikram Shrestha, Yifei Sun, Farzana Faisal, Victoria Kim, Kevin Soares, Alex Blair, Joseph M. Herman, Amol Narang, Avani S. Dholakia, Lauren Rosati, Amy Hacker‐Prietz, Linda Chen, Daniel A. Laheru, Ana De Jesus‐Acosta, Dung T. Le, Ross Donehower, Nilofar Azad, Luis A. Diaz, Adrian Murphy, Valerie Lee, Elliot K. Fishman, Ralph H. Hruban, Tingbo Liang, John L. Cameron, Martin Makary, Matthew J. Weiss, Nita Ahuja, Jin He, Christopher L. Wolfgang, Chiung‐Yu Huang, Lei Zheng

**Affiliations:** ^1^ Department of Oncology The Johns Hopkins University School of Medicine Baltimore Maryland; ^2^ Division of Biostatistics and Bioinformatics Sidney Kimmel Comprehensive Cancer Center The Johns Hopkins University School of Medicine Baltimore Maryland; ^3^ Department of Surgery The Johns Hopkins University School of Medicine Baltimore Maryland; ^4^ Department of Radiation Oncology The Johns Hopkins University School of Medicine Baltimore Maryland; ^5^ Department of Radiology The Johns Hopkins University School of Medicine Baltimore Maryland; ^6^ The Sol Goldman Pancreatic Cancer Research Center Department of Pathology The Johns Hopkins University School of Medicine Baltimore Maryland; ^7^ Sidney Kimmel Comprehensive Cancer Center The Johns Hopkins University School of Medicine Baltimore Maryland; ^8^ Department of Biostatistics Johns Hopkins Bloomberg School of Public Health Baltimore Maryland; ^9^ The Second Affiliated Hospital of Zhejiang University Hangzhou China; ^10^ Department of Radiation Oncology M.D. Anderson Cancer Center Houston Texas

**Keywords:** Borderline resectable pancreatic adenocarcinoma, chemoradiation, neoadjuvant therapy, pancreatic cancer, resectability

## Abstract

The use of neoadjuvant chemotherapy or radiation for borderline resectable pancreatic adenocarcinoma (BL‐PDAC) is increasing. However, the impact of neoadjuvant chemotherapy and radiation therapy on the outcome of BL‐PDAC remains to be elucidated. We performed a retrospective analysis of 93 consecutive patients who were diagnosed with BL‐PDAC and primarily followed at Johns Hopkins Hospital between February 2007 and December 2012. Among 93 patients, 62% received upfront neoadjuvant chemotherapy followed by chemoradiation, whereas 20% received neoadjuvant chemoradiation alone and 15% neoadjuvant chemotherapy alone. Resectability following all neoadjuvant therapy was 44%. Patients who underwent resection with a curative intent had a median overall survival (mOS) of 25.8 months, whereas those who did not undergo surgery had a mOS of 11.9 months. However, resectability and overall survival were not significantly different between the three types of neoadjuvant therapy. Nevertheless, 22% (95% CI, 0.13–0.36) of the 58 patients who received upfront chemotherapy followed by chemoradiation remained alive for a minimum of 48 months compared to none of the 19 patients who received upfront chemoradiation. Among patients who underwent curative surgical resection, 32% (95% CI, 0.19–0.55) of those who received upfront chemotherapy remained disease free at least 48 months following surgical resection, whereas none of the eight patients who received upfront chemoradiation remained disease free beyond 24 months following surgical resection. Neoadjuvant therapy with upfront chemotherapy may result in long‐term survival in a subpopulation of patients with BL‐PDAC.

## Introduction

Surgery remains the only potentially curative treatment for pancreatic ductal adenocarcinoma (PDAC). However, only 15–20% of patients with newly diagnosed PDAC are eligible for potentially curative surgical resection 1, 2. Therefore, increasing the proportion of patients who are eligible for curative resection is a potential strategy to improve the overall outcomes of PDAC. Among the patients who are potential surgical candidates, some are considered to have a “borderline resectable PDAC (BL‐PDAC).” The most well‐established, CT‐based classification of BL‐PDAC was developed at the M. D. Anderson Cancer Center (MDACC). This classification was broadly accepted at the consensus conference of the American Hepato‐Pancreato‐Biliary Association (AHPBA), the Society of Surgical Oncology (SSO), and the Society of the Surgery of Alimentary Tract (SSAT) in 2009 3, and has been incorporated into the National Comprehensive Cancer Network (NCCN) guidelines 4 to include the following scenarios of blood vessel involvement:

- CT findings of venous distortion of the superior mesentery vein (SMV)/portal venous (PV) axis including short‐segment venous occlusion with sufficient proximal and distal vessel length to allow safe reconstruction;
- Encasement of the gastroduodenal artery up to the common hepatic artery (CHA), with either short‐segment encasement or direct abutment of the CHA without extension to the celiac axis (CA);
- Tumor abutment of the superior mesentery artery (SMA) or CA, but with no greater than 180° of the vessel wall circumference.

Although BL‐PDAC is technically resectable, the rate of microscopic margin‐positive (R1) resection is high 5. It has been well recognized that R1 resections are associated with inferior survival compared to margin‐negative (R0) resections 1. Therefore, neoadjuvant chemotherapy and radiation are being used more commonly for BL‐PDAC 5, 6, 7, 8, 9; however, their impact on the outcomes and resectability of BL‐PDAC is not well‐established. Several institutional retrospective studies have suggested that neoadjuvant therapy for BL‐PDAC is associated with survival benefit 5, 7, 8, 9. However, not all patients who received neoadjuvant therapy are able to undergo a potentially curative resection 6. Moreover, none of these studies reported on consecutive patients who initially presented with BL‐PDAC. In addition, there are limited data describing follow‐up on those patients who received neoadjuvant therapy with intent for later resection but who did not proceed to resection. Therefore, we performed an intention‐to‐treat analysis of consecutive patients who were diagnosed with BL‐PDAC and primarily followed at our institution.

## Methods

### Study design

Consecutive patients with PDAC who were seen by a physician at Johns Hopkins Hospital (JHH) between February 2007 and December 2012 were reviewed retrospectively. Ninety‐three patients were identified who met the following criteria: (1) treated and/or primarily followed at JHH with neoadjuvant therapy and/ or surgery; (2) met the diagnostic criteria of BL‐PDAC as per the SSO/AHPBA/SSAT/NCCN guidelines; and (3) evaluated at the JHH Pancreatic Cancer Multidisciplinary Clinic or by a similar multidisciplinary team prior to the initiation of treatment. Diagnoses of BL‐PDAC were documented on clinic notes following multidisciplinary evaluation and later confirmed by reviewing the vessel involvement information retrieved from CT radiology reports from E.K.F. Patients with carcinomas that abutted the PV/SMV without vein distortion and without arterial involvement were excluded as those cases are considered to be resectable at JHH. Following neoadjuvant therapy, the resectability was determined as a consensus at the multidisciplinary tumor board. Diagnostic laparoscopy is not routinely used at JHH. All surgeries were performed by high‐volume surgeons (>50 cases every year).

### Statistical analysis

Univariate and multivariate logistic regressions were performed to examine the association between resectability of BL‐PDAC and other potential risk factors, including baseline clinicopathologic factors and initial therapy. Unadjusted and adjusted odds ratios (OR) were reported with 95% confidence intervals. Overall survival (OS) was defined as the time from the onset of initial therapy to death due to any cause. For patients who underwent surgery, recurrence‐free survival (RFS) was calculated from surgery to disease recurrence or death, whichever occurred first. For patients who did not undergo surgery, progression‐free survival (PFS) was defined as the time from the initiation of neoadjuvant therapy to disease progression by radiological imaging or death, whichever occurred first. For all time‐to‐event analyses, the date of administrative censoring was May 15, 2015. Median OS, RFS, and PFS were estimated using the Kaplan–Meier method, and were compared between subgroups using log‐rank tests. Univariate and multivariate Cox proportional hazards regression were used to evaluate effect of baseline clinicopathologic factors including age, gender, performance status, CA19‐9, vessel involvement, and initial therapy on the risk of experiencing events. Surgical resection margin status and lymph node involvement were not involved in univariate or multivariate analysis because the majority of patients had negative margin and no lymph node involvement following neoadjuvant therapy. Unadjusted and adjusted hazard ratios (HR) were reported with 95% confidence intervals. All *P*‐values are two‐sided and *P* < 0.05 was considered as statistically significant. Statistical analysis was performed using R software, version 3.3.0 (The R Foundation for Statistical Computing).

## Results

### Clinicopathologic characteristics

Demographic and clinical characteristics of the 93 patients with BL‐PDAC are summarized in Table 1. The median age of the study cohort was 65 years (range, 42–83 years). There were 39 females and 54 males. The majority of patients (95%) had a baseline ECOG performance status of 0‐1. Serum carbohydrate antigen 19‐9 (CA 19‐9) was measured at the initial clinic visit: 69 patients (74%) had a CA 19‐9 ≤1000 U/mL and 18 patients (19%) had CA 19‐9 >1000 U/mL. The remaining six patients (7%) did not have CA 19‐9 levels recorded at the initial visit.

**Table 1:** Patient characteristics at baseline and initial therapies

Characteristic	*n* = 93	Chemo (*n* = 14)	Chemorad (*n* = 19)	Comb (*n* = 58)
Sex‐ *n* (%)				
Female	39 (41.9)	8	10	20
Male	54 (58.1)	6	9	38
Age, years – *n* (%)				
Median	65			
Range	42‐83			
ECOG performance status – *n* (%)				
0	53 (57.0)	7	9	37
1	34 (36.6)	5	10	19
2	3 (3.2)	1	0	2
3	1 (1.1)	1	0	0
CA 19‐9, U/mL – *n* (%)				
≤1000	69 (74.2)	11	12	45
>1000	18 (19.4)	3	5	9
Missing	6 (6.5)			
Vessel involvement – *n* (%)				
Venous only	45 (48.4)	10	11	23
Arterial only	15 (16.1)	2	1	11
Both venous and arterial	33 (35.5)	2	7	24
Initial therapy – *n* (%)				
Chemotherapy only	14 (15.1)			
Chemoradiation only	19 (20.4)			
Chemotherapy > chemoradiation	58 (62.4)			
Surgery	2 (2.2)			

**Figure 1:**
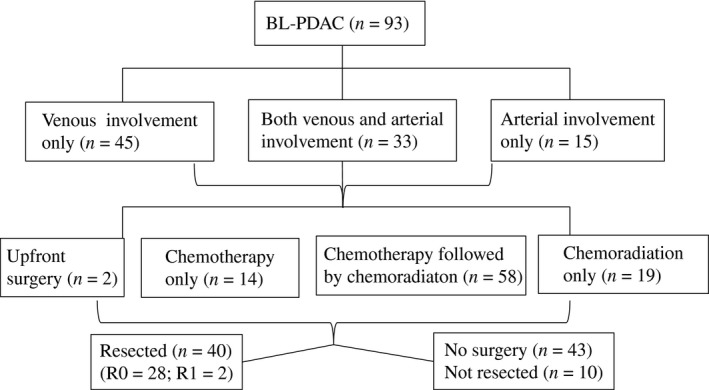
Flowchart of patient characteristics.

All patients were evaluated by pancreatic protocol CT with 3D reconstruction. Among them, 45 patients’ tumors (48%) had isolated venous involvement of the PV/SMV; 15 patients’ tumors (16%) involved abutment of one or more of the arteries including CA, SMA, or CHA, or short‐segment encasement of CHA; and 33 patients’ tumors (35%) had both venous and arterial involvement (Fig. 1).

The majority (98%) of patients in this cohort received neoadjuvant therapy: 19 patients (20%) received upfront chemoradiation where chemotherapy was administrated as a radio‐sensitizer; 58 patients (62%) received upfront chemotherapy followed by chemoradiation; and 14 patients (15%) received chemotherapy only. The remaining two patients opted for upfront surgery without neoadjuvant therapy and were excluded from the statistical analyses unless otherwise indicated. Chemoradiation regimens varied with most ranging from 45‐54 Gy in 28–30 fractions administered concurrently with either infusional 5‐fluorouracil, capecitabine, or gemcitabine. Among the 72 patients who had received chemotherapy as a part of neoadjuvant therapy excluding chemotherapy as a radio‐sensitizer, 56 patients (78%) received a gemcitabine‐based regimen; 13 patients (18%) received a FOLFOX/FOLFIRINOX‐based regimen (infusional 5‐fluorouracil, oxaliplatin and/irinotecan); and 3 patients (4%) received both types of chemotherapies sequentially. The duration of chemotherapy from the initiation of treatment until the patients either underwent surgical exploration or were deemed to be no longer candidates for surgical resection had a wide range from one to nine cycles with 3–4 weeks per each cycle with a median duration of approximately three cycles. There is no obvious difference in the choices of neoadjuvant chemotherapy regimens and the duration of chemotherapy between upfront chemotherapy alone and upfront chemotherapy followed by chemoradiation. Among those patients who underwent surgical resection, the median duration of neoadjuvant chemotherapy was approximately two cycles.

### Clinical outcomes

The median follow‐up for 91 patients, excluding the 2 patients who underwent upfront surgical resection, was 15.1 months (range, 2.7–87.5). The median overall survival of these 91 patients was 15.1 months. Among them, 40 patients had their tumors resected with a curative intent; and thus the resectability was 44%. At the time of last follow‐up, there were 12 patients (13%) alive with a median follow‐up of 53.2 months (range, 32.7–87.5). Eight of the 12 (67%) surviving patients did not have evidence of disease.

A total of 50 patients completed neoadjuvant treatment without radiographic progression and were offered surgical resection with curative intent; however, 10 (20%) patients were found intraoperatively to have unresectable or metastatic disease and surgery was, therefore, aborted (Fig. 1). No patients were excluded from resection before or during laparotomy due to toxicity of neoadjuvant therapy. Out of the 40 patients who underwent curative surgical resection, including two patients who underwent upfront surgical resection, 95% achieved a margin‐negative (R0) resection and 70% had no regional lymph node metastasis. The remaining 43 patients did not undergo curative surgery due to local disease progression and/or the development of metastatic diseases during the course of neoadjuvant therapies.

### Lack of predictive characteristics for resectability of BL‐PDAC

First, we attempted to identify if there were clinical factors at the time of diagnosis or types of neoadjuvant therapy that would affect resectability using univariate and multivariate logistic regression (Table S1; Table 2). Including the two patients who underwent upfront surgical resection, 25 (58%) of the 45 patients with isolated venous involvement were found to be resectable. Of the 15 patients with isolated arterial involvement, 10 (67%) were found to be resectable; and, 14 (42%) of the 33 patients with both arterial and venous involvement were resectable.

**Table 2:** Multivariate logistic regression analysis of the factors affecting probability of resectability

Risk factor	Odds ratio	95% CI	*P*
Gender			
Female	1 (Ref)	–	–
Male	0.91	0.35–2.37	0.85
Age			
≤65	1 (Ref)	–	–
>65	0.49	0.19–1.27	0.14
ECOG			
0	1 (Ref)	–	–
≥1	0.51	0.19–1.35	0.17
CA 19‐9			
<1000	1 (Ref)	–	–
≥1000	0.83	0.27–2.56	0.74
Vessel involvement			
Venous only	1 (Ref)	–	–
Arterial only	1.52	0.36–6.36	0.57
Both arterial and venous	0.37	0.13–1.06	0.06
Neoadjuvant therapy			
Chemoradiation only	1 (Ref)	–	–
Chemo only	0.64	0.14–2.98	0.57
Chemo>chemoradiation	1.12	0.33–3.78	0.86

For all of the logistic regression analyses below, the two patients who underwent upfront surgical resection were excluded. Shown in Table 2, none of the baseline clinicopathologic factors including the types of vessel involvement were significantly associated with increased or decreased resectability. Adjusting for other baseline clinicopathologic factors, BL‐PDACs with both arterial and venous involvement had marginally significant lower odds of resectability than those involving vein only (OR, 0.37; 95% CI, 0.13–1.06; *P* = 0.06). Compared to chemoradiation therapy alone, neither chemotherapy alone (OR, 0.64; 95% CI, 0.14–2.98) nor upfront chemotherapy followed by chemoradiation (OR, 1.12; 95% CI, 0.33–3.78) were significantly associated with resectability. Thus, we were not able to identify predictive characteristics for resectability of BL‐PDAC.

### Upfront chemotherapy followed by chemoradiation was significantly associated with longer progression‐free survival in nonsurgical BL‐PDAC patients

We then analyzed patients who did not undergo surgery due to either the development of metastases or local disease progression to an obviously unresectable condition (Table S2; Table 3). Among these 43 patients, median PFS was 4.3 months. Of the clinical factors analyzed in the multivariate Cox regression (Table 3), upfront chemotherapy followed by chemoradiation was associated with a better PFS than chemotherapy alone (HR, 0.53; 95% CI, 0.21–1.36). However, this result is not statistically significant and could be attributed to the fact that if the patients developed disease progression during the initial course of chemotherapy, they would not have a chance of receiving chemoradiation. ECOG ≥ 1, male gender, and age of ≤65 were associated with inferior PFS. Interestingly, BL‐PDACs with both arterial and venous involvement had a significantly better outcome than those involving vein only (HR, 0.34; 95% CI, 0.15–0.78). Even BL‐PDAC with isolated arterial involvement had a strong trend toward better PFS than those with isolated venous involvement (HR, 0.31; 95% CI, 0.09–1.09). It remains possible that there are other risk factors that are not accounted for in this analysis.

**Table 3:** Multivariate Cox regression analysis of factors affecting progression‐free survival among patients who did not undergo surgery

Risk factor	Hazard ratio	95% CI	*P*
Gender			
Female	1 (Ref)	–	–
Mal	4.06	1.72–9.63	<0.01
Age			
≤65	1 (Ref)	–	–
>65	0.32	0.13–0.77	0.01
ECOG			
0	1 (Ref)	–	–
≥1	2.69	1.18–6.17	0.02
CA 19‐9			
<1000	1 (Ref)	–	–
≥1000	1.60	0.63–4.05	0.32
Vessel involvement			
Venous only	1 (Ref)	–	–
Arterial only	0.31	0.09–1.09	0.07
Both arterial and venous	0.34	0.15–0.78	0.01
Neoadjuvant therapy			
Chemoradiation only	1 (Ref)	–	–
Chemo only	2.09	0.66–6.67	0.21
Chemo > chemoradiation	0.53	0.21–1.36	0.18

### Long‐term survival benefit with upfront chemotherapy

**Figure 2:**
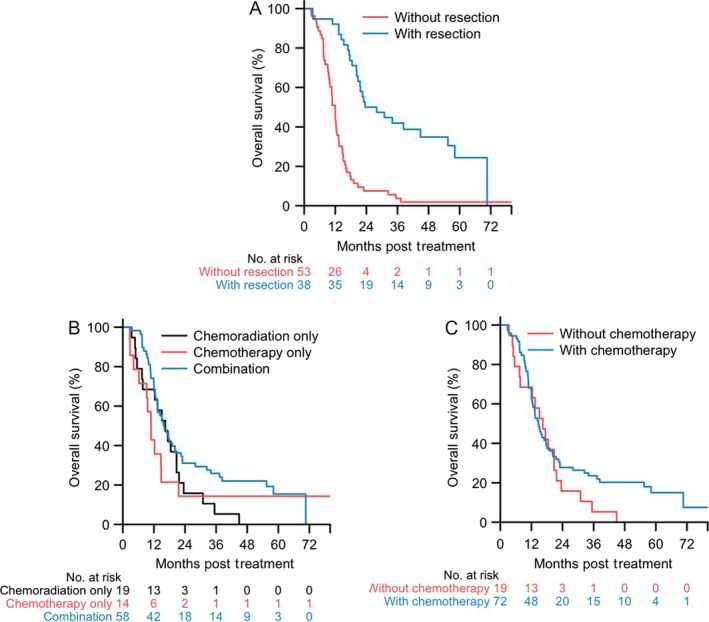
Overall survival of BL‐PDAC. (A) Kaplan–Meier curves for overall survival of BL‐PDAC with (*n* = 40) and without curative surgical resection (*n* = 53), respectively (log‐rank test, *P*< 0.0001). (B) Kaplan–Meier curves for overall survival of BL‐PDAC with upfront chemotherapy only (*n* = 14), upfront chemoradiation only (*n* = 19), and upfront chemotherapy followed by chemoradiation (Combination; *n* = 58), respectively (log‐rank test, *P* = 0.15). (C) Kaplan–Meier curves for overall survival of all BL‐PDACs with upfront chemotherapy either alone or followed by chemoradiation (With chemotherapy; *n* = 72) and with upfront chemoradiation or upfront surgery (Without chemotherapy; *n* = 19), respectively (log‐rank test, *P* = 0.21).

Next, we examined the factors that could have an impact on OS in all patients with BL‐PDAC in both univariate and multivariate Cox regression analysis (Table S3; Table 4). As anticipated, patients who underwent curative resection had a significantly longer OS than those who did not (25.8 vs. 11.9 months; *P* < 0.0001) (Fig. 2A).

**Table 4:** Multivariate Cox regression analysis of factors affecting overall survival

Risk factor	Hazard ratio	95% CI	*P*
Gender			
Female	1 (Ref)	–	–
Male	1.46	0.87–2.43	0.15
Age			
≤65	1 (Ref)	–	–
>65	0.85	0.50–1.42	0.53
ECOG			
0	1 (Ref)	–	–
≥1	1.73	1.01–2.96	0.04
CA 19‐9			
<1000	1 (Ref)	–	–
≥1000	1.19	0.64–2.21	0.58
Vessel involvement			
Venous only	1 (Ref)	–	–
Arterial only	1.48	0.71–3.08	0.29
Both arterial and venous	0.71	0.41–1.22	0.21
Neoadjuvant therapy			
Chemoradiation only	1 (Ref)	–	–
Chemo only	0.73	0.30–1.77	0.48
Chemo>chemoradiation	0.63	0.32–1.26	0.19
Surgical resection			
No	1 (Ref)	–	–
Yes	0.19	0.11–0.34	<0.0001

Among patients receiving upfront chemotherapy followed by chemoradiation, those receiving chemotherapy only, and those receiving upfront chemoradiation alone showed a median OS of 15.9, 10.9, and 16.4 months, respectively (Fig. 2B). In the univariate Cox regression analysis of prognostic factors for OS, upfront chemotherapy was not better than chemoradiation alone (Table S3). It should be noted that in the multivariate Cox regression, both chemotherapy alone (HR 0.73; 95% CI, 0.30–1.77), and upfront chemotherapy followed by chemoradiation (HR, 0.63; 95% CI, 0.32–1.26) were associated with improved survival in comparison to the upfront chemoradiation patients, but the associations were not statistically significant likely due to small sample sizes. Nevertheless, it should be noted that from the date of first treatment initiation, as many as 22% (95% CI, 0.13–0.36) of the 58 patients who received upfront chemotherapy followed by chemoradiation remained alive for a minimum of 48 months compared to none of 19 patients who received upfront chemoradiation (Fig. 2B). Similarly, upfront chemotherapy either alone or followed by chemoradiation, but not upfront local therapies including either chemoradiation or surgery may also be associated with extended survival (Fig. 2C). These results suggest that upfront chemotherapy may make a subpopulation of BL‐PDAC achieve a relatively long‐term survival.

### Upfront chemotherapy may be associated with long‐term disease‐free survival in BL‐PDAC following curative resection

**Figure 3:**
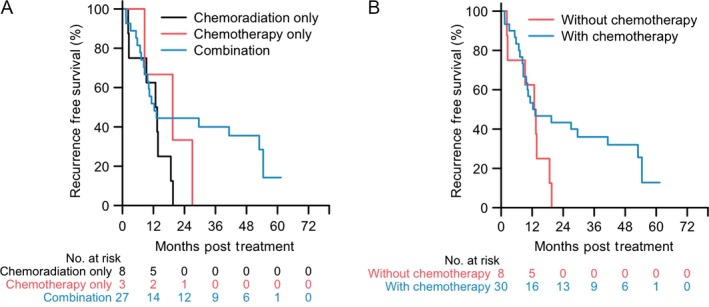
Recurrence‐free survival of BL‐PDAC patients who underwent curative resection. (A) Kaplan–Meier curves comparing upfront chemotherapy followed by chemoradiation (Combination; *n* = 27), upfront chemotherapy only (*n* = 3), and upfront chemoradiation only (*n* = 8), respectively (log‐rank test, *P* = 0.11). (B) Kaplan–Meier curves comparing upfront chemotherapy either alone or followed by chemoradiation (With chemotherapy; *n* = 30) and upfront chemoradiation (Without chemotherapy; *n* = 8), respectively (log‐rank test, *P* = 0.23).

Median RFS among patients who had surgical resection with a curative intent, excluding the two patients who underwent upfront surgical resection, was 13.0 months. Three patients had local recurrence, whereas all other patients developed distant metastases. All the three patients who had local recurrence received upfront chemotherapy followed by chemoradiation. As shown by the univariate and multivariate Cox regression analysis (Table S4; Table 5), upfront chemotherapy followed by chemoradiation may be associated with a better RFS when compared to chemoradiation alone, (HR, 0.44; 95% CI, 0.12–1.53) although the differences did not reach statistical significance likely due to the small sample sizes. In patients who underwent curative surgical resection, if calculated from the date of treatment initiation, the median time to recurrence following upfront neoadjuvant chemotherapy, including three patients who received upfront chemotherapy only, was 19.9 months (Fig. 3B). More importantly, approximately 32% (95% CI, 0.19–0.55) of patients who received upfront chemotherapy remained disease‐free at a minimum of 48 months following surgical resection, whereas none of the eight patients who received upfront chemoradiation remained disease‐free beyond 24 months following surgical resection (Fig. 3B). These results suggest that neoadjuvant therapy with upfront chemotherapy may result in extended recurrence‐free survival in a subpopulation of BL‐PDACs following surgical resection. It may also suggest that upfront chemoradiation delays full‐dose chemotherapy, allowing systemic progression.

**Table 5:** Multivariate analysis of factors associated with recurrence‐free survival among patients who underwent curative surgical resection

Risk factor	Hazard ratio	95% CI	*P*
Gender			
Female	1 (Ref)	–	–
Male	1.75	0.60–5.05	0.30
Age			
≤65	1 (Ref)	–	–
>65	1.21	0.51–2.87	0.66
ECOG			
0	1 (Ref)	–	–
≥1	1.45	0.57–3.72	0.43
CA 19‐9			
<1000	1 (Ref)	–	–
≥1000	1.32	0.45–3.90	0.61
Vessel involvement			
Venous only	1 (Ref)	–	–
Arterial only	0.74	0.25–2.26	0.60
Both arterial and venous	0.53	0.23–1.77	0.39
Neoadjuvant therapy			
Chemoradiation only	1 (Ref)	–	–
Chemo only	0.44	0.08–2.40	0.34
Chemo>chemoradiation	0.44	0.12–1.53	0.19

## Discussion

As the Johns Hopkins Hospital is one of the largest centers in the United States for pancreatic cancer diagnosis and treatment, it is important to report its experience in managing BL‐PDAC. In this study, we found that neoadjuvant therapy with upfront chemotherapy resulted in long‐term survival in a subpopulation of patients with BL‐PDAC. This was not only implicated in patients who underwent curative resection, but also in patients who did not undergo surgery due to disease progression. These results highlight the importance of systemic control in the management of PDAC.

Although upfront neoadjuvant chemotherapy for BL‐PDAC has already been adopted by many institutions, evidence supporting such a management strategy has been scarce. Previously, a retrospective study in BL‐PDAC had demonstrated that chemotherapy followed by chemoradiation therapy is superior to chemoradiation followed by chemotherapy 10. With a retrospective intention‐to treat analysis, Rose et al. reported their cohort of 64 patients with BL‐PDAC who received 24‐week neoadjuvant therapy. In that study results were similar to our observations: 31 (48%) patients underwent resection; with 27 (87%) having a R0 resection. The median OS durations of 23.6 months in all 64 patients and 15.4 months in unresectable patients are longer than those in our cohort, suggesting that prolonged neoadjuvant therapy are potentially beneficial. Long‐term outcomes were not studied by Rose et al. Our study is thus the first retrospective intention‐to‐treat analysis to examine the impact of upfront chemotherapy on the long‐term survival outcome among a relatively large sample size of patients with BL‐PDAC.

The first large retrospective study of BL‐PDAC was published by investigators at the M.D. Anderson Cancer Center in 2007 6. The outcomes of patients with BL‐PDAC in our study are similar to those in this previously published study. Most recent published studies of BL‐PDAC do not include the patients who received neoadjuvant therapy with intent for later resection but did not successfully proceed to resection. Thus, these studies may have selected patients who have a favorable outcome and would more likely benefit from an operation 5, 7, 8, 9. Our study is one of the largest cohorts of BL‐PDAC, and included all consecutive patients treated and primarily followed at our institution. The outcome of the patients who were considered to be candidates for surgical resection is anticipated to be better than the entire cohort of patients with BL‐PDAC. Contemporary chemotherapy regimens such as FOLFIRINOX 11 or the combination of gemcitabine and nab‐paclitaxel 12 started to be used after 2009 and 2013, respectively and thus were not used in a majority of patients in our study which may contribute to the worse OS of our patients than that reported in more recent studies. Another explanation is the short duration of neoadjuvant chemotherapy (two cycles as the median duration) in our cohort of the patients. Longer duration of neoadjuvant therapy may help select patients BL‐PDAC with a better biology for surgical resection. Whether longer duration of neoadjuvant therapy would truly change the outcome of the surgery itself warrants further investigation.

The resectability of BL‐PDAC following neoadjuvant therapies reported in our study was 44% and is comparable to the resectability reported in the M.D. Anderson study 13. The concern in missing the opportunity for curative resection during the course of neoadjuvant therapy still exists. Strategies to predict the resectability of BL‐PDAC at the time in which therapy is initiated also remains unclear. CT scan has been routinely used to reevaluate the resectability following the neoadjuvant therapy. However, several prior studies including one from our institution showed that postneoadjuvant therapy radiographic changes do not predict resectability 14, 15. Because BL‐PDAC is technically resectable, unless there is radiographic evidence of disease progression, patients should undergo laparotomy even without a radiographic or CA19‐9 response. In this study, we also fail to identify any clinical factors or radiographic findings that may predict resectability. Interestingly, our study found that isolated venous involvement is associated with worse outcome than isolated arterial involvement. Because pancreatectomy with vein resection was shown to have the same outcome as pancreatectomy without 16, the use of neoadjuvant therapy in cases of isolated venous involvement remains debated. In our study, patients with isolated venous involvement received similar neoadjuvant therapies as those with other types of vessel involvement. The worse outcome of isolated venous involvement cannot be explained by any unique biology underlying venous involvement as patients with both arterial and venous involvement also were superior to those with isolated venous involvement. Ultimately, such results may not be meaningful due to the small sample size. The effects of different types of vessel involvement and neoadjuvant therapies on BL‐PDAC outcomes remain to be examined with prospective, multi‐center studies in a larger sample size.

In this study, we also did not observe an effect of baseline CA19‐9 and performance status on clinical outcomes, likely attributed again to the small sample size. We also did not analyze the effect of adjuvant therapy due to the limited numbers in our study population. At the time when patients in this cohort were treated, our institution had not started to routinely report the pathologic response following neoadjuvant therapy. CA19‐9 response was also not routinely assessed. It will be interesting to see in the future whether pathologic response or CA19‐9 response would influence clinical outcomes. Furthermore, the CA19‐9 response's impact on predicting resectability will also warrant additional investigation.

Although this study has a relatively large sample size of BL‐PDAC patient population compared to most of the published studies, we recognized we still did not have sufficient power to detect a meaningful effect size. However, in the time‐to‐event analyses, a great proportion of patients experienced the event of interest. For overall survival, we observed 79 deaths among the 91 patients. All of the 43 patients who did not undergo surgery had a PFS event. Moreover, among the 38 patients who underwent surgery and were margin‐negative, 30 of them had recurrence‐free survival event. We included the important risk factors in the regression models to account for potential confounders. Fisher's exact test was performed to examine the pairwise associations between risk factors considered in the regression models. All of the associations were not statistically significant, except that older age (> 65 years) was significantly associated with a higher ECOG score (≥1) (*P* = 0.02). As any single institutional study would be limited by its sample size for this particular patient population, a multi‐institutional study to combine patients from different institutions is warranted.

Chemoradiation given over 5–6 weeks can also delay full‐dose chemotherapy resulting in earlier systemic progression and inferior survival. Recently, stereotactic body radiation therapy (SBRT) has been found to be an effective alternative to conventional chemoradiation in patients with borderline and unresectable pancreatic cancer as reported by Moffitt, Stanford, and JHH 17, 18, 19, 20. SBRT can be given in only 3–5 days with limited toxicity and, therefore, does not delay full‐dose systemic therapy. However, unlike a randomized trial, observational studies like this current one are limited by the fact that many different types of chemotherapy regimens were chosen by medical oncologists likely with the consideration of patient factors and not assigned at random. Randomized studies such as the new Alliance study for patients with BL‐PDAC will evaluate the role of SBRT by randomizing patients to FOLFIRNOX alone or FOLFIRNOX followed by SBRT and then surgery 21. This study should provide important insight into whether SBRT improves pathologic and clinical outcomes.

## Conclusions

This retrospective study supports the use of neoadjuvant therapy with upfront chemotherapy for the treatment of BL‐PDAC. However, since this is an observational study, there can be confounders that are not accounted for in the data analysis. Although the sample size in this study is relatively large for the BL‐PDAC patient population compared to most of the published studies, it is still limited and may contribute to many of the nonsignificant findings reported. Nevertheless, prospective, randomized studies are warranted to establish the role of neoadjuvant therapy in BL‐PDAC 21, 22. The current practice of neoadjuvant therapy needs to be further improved. Whether modern chemotherapy regimens such as FOLFIRINOX and gemcitabine/nab‐paclitaxel and technological advancement with SBRT may further improve the resectability of BL‐PDAC should be investigated.

## Conflict of Interest

The authors declare that they have no competing interests.

## Supporting information


**Table S1.** Univariate logistic regression analysis of factors affecting probability of resectability.
**Table S2.** Univariate Cox regression analysis of factors affecting progression‐free survival among patients who did not undergo surgery.
**Table S3.** Univariate Cox regression analysis of factors associated with overall survival.
**Table S4.** Univariate analysis of factors associated with recurrence‐free survival among patients who underwent curative surgical resection.
