# Supplementary material for: Long‐term survival benefit of upfront chemotherapy in patients with newly diagnosed borderline resectable pancreatic cancer
Source: Cancer Med. 2017 Jun 21;6(7):1552–62. doi: 10.1002/cam4.1104 (PMC5504321; doi:10.1002/cam4.1104)
Supplement: Supplementary file 1 — Table S1. Univariate logistic regression analysis of factors affecting probability of resectability. Table S2. Univariate Cox regression analysis of factors affecting progression‐free survival among patients who did not undergo surgery. Table S3. Univariate Cox regression analysis of factors associated with overall survival. Table S4. Univariate analysis of factors associated with recurrence‐free survival among patients who underwent curative surgical resection. [file CAM4-6-1552-s001.pdf]

Supplemental Tables

Supplemental Table 1 Univariate logistic regression analysis of factors affecting probability of resectability.

Risk Factor	Odds Ratio	95% CI	*P*
**Gender**			
Female	1 (Ref)	–	–
Male	1.42	0.62 to 3.25	0.41
**Age**			
≤ 65	1 (Ref)	–	–
> 65	0.44	0.19 to 1.01	0.05
**ECOG**			
0	1 (Ref)	–	–
≥ 1	0.64	0.28 to 1.47	0.29
**CA 19-9**			
< 1000	1 (Ref)	–	–
≥ 1000	0.92	0.32 to 2.59	0.87
**Vessel involvement**			
Venous only	1 (Ref)	–	–
Arterial only	1.46	0.43 to 4.98	0.54
Both arterial and venous	0.54	0.22 to 1.34	0.18
**Neoadjuvant therapy**			
Chemoradiation only	1 (Ref)	–	–
Chemo only	0.68	0.17 to 2.71	0.58
Chemo > chemoradiation	1.11	0.39 to 3.13	0.85

Supplemental Table 2 Univariate Cox regression analysis of factors affecting progression free survival among patients who did not undergo surgery

Risk Factor	Hazard Ratio	95% CI	*P*
**Gender**			
Female	1 (Ref)	–	–
Male	1.23	0.67 to 2.26	0.51
**Age**			
≤ 65	1 (Ref)	–	–
> 65	0.89	0.47 to 1.66	0.71
**ECOG**			
0	1 (Ref)	–	–
≥ 1	2.19	1.12 to 4.29	0.02
**CA 19-9**			
< 1000	1 (Ref)	–	–
≥ 1000	1.41	0.67 to 2.98	0.37
**Vessel involvement**			
Venous only	1 (Ref)	–	–
Arterial only	0.83	0.30 to 2.26	0.71
Both arterial and venous	0.48	0.24 to 0.97	0.04
**Neoadjuvant therapy**			
Chemoradiation only	1 (Ref)	–	–
Chemo only	0.95	0.36 to 2.51	0.91
Chemo > chemoradiation	0.42	0.19 to 0.96	0.04

Supplemental Table 3 Univariate Cox regression analysis of factors associated with overall survival

Risk Factor	Hazard Ratio	95% CI	*P*
**Gender**			
Female	1 (Ref)	–	–
Male	1.15	0.73 to 1.81	0.56
**Age**			
≤ 65	1 (Ref)	–	–
> 65	1.07	0.69 to 1.66	0.77
**ECOG**			
0	1 (Ref)	–	–
≥ 1	1.52	0.97 to 2.37	0.07
**CA 19-9**			
< 1000	1 (Ref)	–	–
≥ 1000	1.53	0.88 to 2.69	0.14
**Vessel involvement**			
Venous only	1 (Ref)	–	–
Arterial only	1.02	0.54 to 1.93	0.94
Both arterial and venous	0.86	0.53 to 1.41	0.56
**Neoadjuvant therapy**			
Chemoradiation only	1 (Ref)	–	–
Chemo only	1.10	0.53 to 2.29	0.80
Chemo > chemoradiation	0.66	0.39 to 1.14	0.13
**Surgical resection**			
No	1 (Ref)	–	–
Yes	0.27	0.16 to 0.43	<0.0001

Supplemental Table 4 Univariate analysis of factors associated with recurrence free survival among patients who underwent curative surgical resection

Risk Factor	Hazard Ratio	95% CI	*P*
**Gender**			
Female	1 (Ref)	–	–
Male	1.04	0.50 to 2.15	0.93
**Age**			
≤ 65	1 (Ref)	–	–
> 65	1.10	0.54 to 2.26	0.79
**ECOG**			
0	1 (Ref)	–	–
≥ 1	1.16	0.55 to 2.45	0.69
**CA 19-9**			
< 1000	1 (Ref)	–	–
≥ 1000	2.09	0.82 to 5.33	0.12
**Vessel involvement**			
Venous only	1 (Ref)	–	–
Arterial only	0.89	0.36 to 2.16	0.79
Both arterial and venous	0.47	0.18 to 1.21	0.12
**Neoadjuvant therapy**			
Chemoradiation only	1 (Ref)	–	–
Chemo only	0.73	0.19 to 2.78	0.65
Chemo > chemoradiation	0.48	0.20 to 1.15	0.10
